# Buffalo Hospitals

**Published:** 1885-08

**Authors:** 


### Buffalo Hospitals.

We have received the annual reports of the two larger City Hospitals. The Buffalo General Hospital publishes the 26th annual report; the Buffalo Hospital of the Sisters of Charity issues its first. The latter hospital is not only the largest in the city, but also the oldest, and the only explanation we can give for the extraordinary announcement of a first annual report, after thirty-seven years of existence, is, that until recently many members of the Hospital staff have been connected with the rival hospital; possibly the personal interests of some members of the medical staff made it convenient not to invite comparisons between the two hospitals, or it may have been due simply to indifference.

The annual reports show that at the Buffalo General Hospital, during the year ending Dec. 31, 1884, 771 patients were treated with a mortality of 78 or $10^{1}$ per cent.

At the Buffalo Hospital of the Sisters of Charity, during the year ending Nov. 1, 1884, 1042 patients were treated, with a mortality of 48 or $4_{6}$ per cent.

This comparison is hardly a fair one to make, however, as it includes deaths occurring within 48 hours after admission. Such deaths rarely occur at the Sisters' Hospital, as patients severely injured are commonly taken to the "Emergency Hospital," and from there transferred to the Sisters' when advisable.

We are informed that the total number of deaths, including coroners' cases, and those moribund upon admission, at both "The Sisters'" and "The Emergency Hospital," was 67. Even crediting the total number to the Sisters' Hospital, their mortality is only $6^{4}$, markedly less than the Buffalo General's $10^{1}$.

It is interesting to turn to the results of surgical operations as perhaps better indicating the sanitary condition of the two hospitals. The Buffalo General reports 93 operations (excluding boils and abscesses opened, which appear in the report), with nine deaths following operations, as hereinafter indicated.

| | | | | |
|---|---|---|---|---|
| Urethrotomy, external, | 6 | Cases. | 2 | Deaths. |
| Abscess Pericolitic, aspirated, | 1 | " | 1 | " |
| Amputation at Hip, | 1 | " | 1 | " |
| Amputation at Thigh, | 3 | " | 1 | " |
| Extirpation of Cancerous Thyroid, | 1 | " | 1 | " |
| Resection of Half Lower Jaw, | 1 | " | 1 | " |
| Tracheotomy for Membranous Croup, | 1 | " | 1 | " |
| Trephining, | 6 | " | 1 | " |

The Buffalo Hospital of the Sisters of Charity reports 95 operations, with not a single death following an operation wound. We give the list as contained in the report:

| Surgical Operations performed during the year, commencing Nov. 1, 1883, ending Nov. 1, 1884. | Number. | Recovered. | Deaths. |
|---|---|---|---|
| Amputation of Thigh | 1 | 1 | ...... |
| Amputation of Leg | 6 | 6 | ...... |
| Amputation of Shoulder Joint | 1 | 1 | ..... |
| Amputation of Arm | 4 | 4 | ...... |
| Amputation of Fingers | 10 | 10 | ...... |
| Resection of Elbow | 3 | 3 | ...... |
| Tumors Removed | 11 | 11 | ...... |
| Supra-pubic Lithotomy | 2 | 2 | ...... |
| Litholopaxy | 2 | 2 | ...... |
| Double Harelip | 3 | 3 | ...... |
| Single Harelip | 4 | 4 | ...... |
| Club Foot | 3 | 3 | ..... |
| Internal Urethrotomy | 11 | 11 | ...... |
| External Urethrotomy | 3 | 3 | ...... |
| Radical Cure of Hydrocele | 8 | 8 | ..... |
| Circumcision | 6 | 6 | ...... |
| Fistula in Ano | 6 | 6 | ...... |
| Hernia | 5 | 5 | ..... |
| Removal of Necrosed Bone | 5 | 5 | ...... |
| Varicocele | 1 | 1 | ...... |

It is perhaps unfair to make a comparison on a single year's work, nor would we say that the difference in results is due to any want of skill on the part of the surgeons at the Buffalo General Hospital. On the contrary, we are glad to say that the surgical staff is composed of able men. The "Sisters' Hospital" may possibly have had all the "good cases," and the other hospital all the "bad ones." But we believe it is fair to say that a hospital in which, during one year, and that but a short time ago, *twelve consecutive cases of compound fracture died*, is open to suspicion as to its sanitary condition.

The Sisters' Hospital, on the contrary, has every advantage from a sanitary point of view. The building is new, and has been constructed with due regard to hygienic requirements. It is situated on an elevated plot of ground, containing twenty-five acres, and in proximity to the open parks, and distant from the noises and nuisances of the city. Indeed, it has advantages which very few city hospitals can equal. Above, and beyond this, the patients have the benefit of the loving and gentle care of the good Sisters, whose unwearied labors in their life-long devotion to the welfare of the sick, cannot be compared with the services of young women whose attendance is as scholars in a training school, and who, as they gain the necessary experience, are supplanted by a fresh class of aspirants for hospital training as nurses.

We believe, too, that the excellent results attained have been, in a degree, due to the strict antiseptic precautions which are employed in all operations. Sublimated jute, peat, gauze, wood flour, etc., are used as dressings, and it is to this that the chief surgeon of the hospital attributes the gratifying result, that, during the year past, there has not been a single case of septicæmia or erysipelas, and not one case of death from an operation wound.

It is with some regret that we call attention to these comparative results, which, at least during the past year, show so strongly against the Buffalo General Hospital. Institutions founded and maintained in pure benevolence and kindness towards the sick and suffering are, in a measure, above criticism. The noble women who comprise the Ladies' Hospital associations have labored with a diligence and devotion to the interests of the Hospital which commands our admiration, and it is an unpleasant duty to point out that the best possible results are not attained—though through no fault of theirs.

The superior success at the Sisters' Hospital has greatly disturbed certain members of the Buffalo General Hospital staff, and they have even attempted to explain their own deficiencies by the assertion that the report of the Sisters' Hospital was untrue.

Such a charge is not only unjust, but discreditable to the men who make it. It would be more to the purpose for the medical staff to guard the sanitary condition of their hospital; to see to it that the patients receive the most skillful and careful attention; indeed, the ladies who have the oversight of the institution will not be satisfied with results in any way inferior to those of any similar hospital.

A generous rivalry between the two hospitals for the attainment of the lowest rate of mortality, and the greatest benefit to these patients who entrust themselves, or are entrusted, to their care, will result in a positive advantage to the community, and benefit to both of these great institutions; but if, as may reasonably be expected, the succeeding annual reports of the two hospitals show in favor of the Sisters', we hope that if envy and jealousy cannot be restrained, at least the manifestations will not publicly appear, to the discredit of the profession at large.

The Buffalo General Hospital has done, and is doing, a noble work, but it is the bounden duty of its authorities to keep the entire building free from septic poisons, or it will cease to command the confidence and support of the community.

Many able sanitarians have expressed the opinion that hospitals should be of wood, and burned as soon as the first indications of infections appeared, but we believe that by the aid of modern chemistry, even hospitals of solid masonry may be completely and thoroughly purified. The ordinary methods of scrubbing, whitewashing and fumigating have signally failed, but when chemical agents are applied *in sufficient quantity*, entire success may be expected. In the surgical wards of Bellevue Hospital, for instance, some years ago, the mortality reached alarming proportions. The elder Doremus undertook the disinfection and purification of the building, and accomplished the task by generating between *two and three tons* of chlorine gas in the closed wards. It is only by thorough work of this kind that anything effective can be accomplished. We would remind the medical staff of the excellent results attained when, last Summer, all the patients operated on were removed from the hospital and placed in tents. We believe that equally good results are possible in the Winter time when tents cannot be used, and it is the imperative duty of all hospital authorities to keep their buildings so thoroughly disinfected that an operation within the walls shall never mean greatly increased risk of death to the poor patient.

